# Nuclear localization of alpha-synuclein affects the cognitive and motor behavior of mice by inducing DNA damage and abnormal cell cycle of hippocampal neurons

**DOI:** 10.3389/fnmol.2022.1015881

**Published:** 2022-11-10

**Authors:** Yue Pan, Qinglan Zong, Guoxiang Li, Zhengcun Wu, Tingfu Du, Zhangqiong Huang, Ying Zhang, Kaili Ma

**Affiliations:** Institute of Medical Biology, Chinese Academy of Medical Sciences and Peking Union Medical College, Kunming, China

**Keywords:** alpha-synuclein, nuclear localization, hippocampus, DNA damage, cell cycle, Parkinson’s disease

## Abstract

Nuclear accumulation of alpha-synuclein (α-syn) in neurons can promote neurotoxicity, which is considered the key factor in the pathogenesis of synucleinopathy. The damage to hippocampus neurons driven by α-syn pathology is also the potential cause of memory impairment in Parkinson’s disease (PD) patients. In this study, we examined the role of α-syn nuclear translocation in the cognition and motor ability of mice by overexpressing α-syn in cell nuclei in the hippocampus. The results showed that the overexpression of α-syn in nuclei was able to cause significant pathological accumulation of α-syn in the hippocampus, and quickly lead to memory and motor impairments in mice. It might be that nuclear overexpression of α-syn may cause DNA damage of hippocampal neurons, thereby leading to activation and abnormal blocking of cell cycle, and further inducing apoptosis of hippocampal neurons and inflammatory reaction. Meanwhile, the inflammatory reaction further aggravated DNA damage and formed a vicious circle. Therefore, the excessive nuclear translocation of α-syn in hippocampal neurons may be one of the main reasons for cognitive decline in mice.

## Introduction

Alpha-synuclein (α-syn) is a soluble protein encoded by *SNCA* gene, which consists of 140 amino acid residues. It is mainly involved in the maintenance of normal synaptic function and the transportation of synaptic vesicles, but the pathological accumulation of α-syn is closely related to Parkinson’s disease (PD; Wong and Krainc, 2017). A large number of studies have shown that α-syn exists widely in the nucleus (Yu et al., 2007; Zhang et al., 2008; Vivacqua et al., 2009, 2011; Zhong et al., 2010; Schaser et al., 2019). Moreover, α-syn can interact with DNA after entering the cell nucleus, thereby changing the physical and chemical properties of DNA and affecting the transcription balance (Vasudevaraju et al., 2012; Milanese et al., 2018). When α-syn undergoes excessive nuclear translocation, it promotes serine-129 phosphorylation of α-syn and cause the changes in PD-related genes (Davidi et al., 2020), which further suggests that the excessive nuclear translocation of α-syn is closely related to the pathology of PD, but the underlying mechanism is not sufficiently clear.

Clinical imaging studies have attributed the cognitive decline of PD and Lewy body dementia (LBD) to the damage of hippocampal and cortical circuits, and obvious hippocampal atrophy was observed in PD patients (Molano et al., 2010; Calabresi et al., 2013). Previous studies have shown that α-syn has a negative effect on the growth of dendrites of newborn neurons, which provides sufficient evidence for α-syn to participate in the regulation of hippocampal neurogenesis (Regensburger et al., 2014). Our previous studies have shown that overexpression of α-syn in the hippocampus can damage the survival of neurons in adult mice (Du et al., 2021), and nerve cell death and neuron loss caused by activating external and internal cell apoptosis pathways have been considered the main factors leading to cognitive and memory dysfunction (Dickson, 2004). Therefore, inhibiting apoptosis and regulating neurogenesis are expected to be strategies to improve memory impairment in PD patients. Based on this, some researchers have found that neuronal damage and subsequent cognitive impairment are caused by the activation of neurotoxic astrocytes (Santello et al., 2019), but more evidence is needed on the relationship between α-syn accumulation, astrocyte activation, and neuronal apoptosis.

In this study, we injected rAAV2/9 carrying *EGFP*, *EGFP-SNCA*, *EGFP-SNCA*-NLS, and *EGFP-SNCA*-NES into the hippocampus of mice to construct four kinds of mouse models that specifically express α-syn in different subcellular localizations, so as to explore the effect of overexpression of α-syn in the nucleus on the hippocampal function of mice, and further study its molecular mechanism, thereby providing a basis for studying the effect of α-syn on the survival of hippocampal neurons and inducing PD-related symptoms.

## Materials and methods

### Animals

One hundred 6–8-week-old SPF-grade C57BL/6 J male mice were selected (18–25 g; Institute of Medical Biology, Chinese Academy of Medical Science, Kunming, China) and raised in a barrier environment under a 12-h light/dark cycle at constant temperature (20 ± 2°C) and relative humidity of 50–60% (SYXK (Dian) K2021-0001). All animals had free access to water and food with cage cleaning twice a week. All of the mice were allowed 1 week of habituation to the housing conditions before the start of experiments. The protocol was approved by the Experimental Animal Ethic Committee of the Institute of Medical Biology Chinese Academy of Medical Sciences (DWSP202203024), and the experimental process followed the 3R principle to give humane care to animals.

### Stereotaxic surgery

Before the operation, the mice received intraperitoneal injection of a ready-to-use anesthetic containing 1.25% Avertin at a dose of 0.2 ml/10 g, and AAV-EGFP, AAV-EGFP-SNCA, AAV-EGFP-SNCA-NLS, and AAV-EGFP-SNCA-NES were injected into the hippocampus bilaterally with 5 μl Hamilton microinjection. The injection coordinates were as follows: anteroposterior (AP) -2.5 mm, mediolateral (ML) ±2.0 mm, dorsoventral (DV) -2.0 mm. Recombinant adeno-associated virus rAAV2/9, carrying human SNCA, SNCA-NLS, SNCA-NES, and EGFP, was packaged and purified by Taitool Bioscience Company (Shanghai Taitool Bioscience Co., Ltd., China). The final titer was about 1.5 × 1,013 GC/mL and it was frozen at −80°C for later use.

### Behavioral test

#### Rotarod test

The total time was 5 min, where the first 2 min were set to accelerate exercise and the coordination of mice’s limbs was tested. The following 3 min were set to a constant speed exercise to test the physical strength of the mice. The maximum speed was 40 rmp/h. Each mouse was tested three times, at least 3 min apart, and the time when the mouse fell from the rotating rod was recorded.

#### Open-field test

In accordance with the published test methods (Kraeuter et al., 2019a), we tested the spontaneous activity of mice in the spacious environment. The 50 × 50 cm field was divided into two areas, the center area and the periphery area, and the side length of the center area was about half of the periphery area’s length. Before the test, the mice were taken from the mouse room to the test area and allowed to rest for more than 2 h, after adapting to the environment, the test was started. The mice were uniformly put in from the center, and their activities within 5 min were observed. After each test, we wiped the site with 75% alcohol for disinfection and deodorization, and started the next test after the smell dissipated.

#### Y maze

According to the previously published methods (Kraeuter et al., 2019b), the Y maze test was performed to evaluate the short-term memory of mice. During the training period, one arm (novel arm) of the Y maze was closed, and the mice were placed in the maze for free exploration for 10 min from the start arm. After an interval of 1 h, the novel arm was opened, and the mice were placed in the maze for free exploration for 5 min. The times of entering the novel arm and other arms were recorded and compared to evaluate the degree of spatial memory.

#### Novel object recognition

The novel-object recognition test was carried out in line with the previous methods (Leger et al., 2013) to evaluate the memory of each mouse for object recognition. In the training stage, the mice were placed in the field where two identical objects had been placed in advance to explore freely for 10 min, and then they became familiar with the objects. After an interval of 1 h, one of the old objects was replaced by a new object and the mice were allowed to explore freely for 5 min. We calculated the recognition index to evaluate short-term memory as follows: recognition index = new object exploration time/ (new object exploration time + old object exploration time).

#### Barnes maze

In line with the published methods (Pitts, 2018), the Barnes maze was used to evaluate the learning ability and long-term memory of the mice. After the experiment, we turned on the light above the platform and let the mice explore freely. We allowed the mice escaped from the open platform surface to the dark concave room (target box) below the platform, and kept them there 30 s. They were trained continuously for 5 days, and we recorded the time and error times of their entering the target box (every time the mouse pokes its head into the non-target box, with the eyes below the platform surface, was recorded as an error). We removed the target box on the 3rd day (total 8 days) and 7th day (total 12 days) after training, recorded the time of the mice’s activities in the quadrant where the target box was located within 4 min, and checked the memory retention.

#### Sucrose consumption test

SCT was performed referring to previously published methods on mice that were fed separately (El Yacoubi et al., 2003; Duric et al., 2010), and the depression-like behavior of the mice was observed. The mice adapted to 1% sucrose solution for 48 h before starting the experiment, and were fasted and water prohibited for 12 h before the test. The sucrose solution consumption within 1 h was calculated by weighing the sucrose solution before and after consumption, and the following formula was used: sucrose preference (%) = sucrose solution consumption/ (sucrose solution consumption + normal drinking water consumption).

### 5-Bromo-2′-deoxyuridine staining

The mice received intraperitoneal injection of BrdU (Sigma, B5002; 10 mg/ml) at months 1, 3, and 6 after stereotaxic surgery. For the proliferation study, BrdU (100 mg/kg, i.p.) was injected twice at an interval of 6 h, then perfused from heart with phosphate buffer saline (PBS) and 4% paraformaldehyde after 24 h and fixed in 4% paraformaldehyde for 48 h. In the other group, BrdU (50 mg/kg, i.p.) was injected once a day for five consecutive days at month 1 after stereotaxic surgery to detect the survival rate of neurons. The mice were sacrificed on the third day (survival 3 days) and 21st day (survival 3 weeks) from the last injection of BrdU.

### Western blot analyses

The mice were sacrificed, and their hippocampus was separated and placed in an icy EP tube. RIPA lysis buffer (CW2333, CWBIO, and China) supplemented with 2 mM PMSF and protease inhibitor cocktail (539,131, Millipore, and USA) was added to separate protein samples. We centrifuged the mixture, and the supernatant was taken out for protein quantification with BCA Protein Assay Kit (CW0014S, CEBIO, and China). Then, SDS-PAGE Sample Loading Buffer (P0015L, Beyotime, China) was added and the system was boiled for 5 min. We separated the system on Criterion TGX Stain-Free gels (Bio-Rad) for 120 min at 85 V. After transfer, the membranes were blocked with 5% nonfat milk buffer for 45 min at room temperature, incubated with primary antibodies at 4°C overnight, and then incubated with goat anti-mouse or goat anti-rabbit secondary antibodies at room temperature for 1 h. Later, photo graphs were taken with Odyssey infrared laser scanning, and quantified using Odyssey software.

### Immunofluorescence

The mice were anesthetized with an anesthetic containing 1.25% Avertin, and then perfused with PBS and 4% paraformaldehyde solution. The brain was fixed in 4% paraformaldehyde solution overnight, and dehydrated by a dehydrator. After embedding, we cut slices with a thickness of 4 μm. The slices were baked in an oven at 65°C, and then dewaxed and hydrated by conventional method. The samples were boiled in Tris-EDTA buffer for 15 min in microwave oven for antigen repair. After cooling to room temperature, incubated with goat serum for 1 h, and the corresponding diluted primary antibody was dripped and incubated overnight at 4°C. After washing with PBS, rabbit or mouse fluorescent secondary antibodies were dripped. After DAPI (Abcam, ab104139) sealing, images were captured by Panoramic MIDI.

### TUNEL staining

Apoptosis in the hippocampus was determined using the terminal deoxynucleotidyl transferase-mediated YF®594-dUTP nick-end labeling (TUNEL) method following the manufacturer’s protocol (YF®594 TUNEL Assay Apoptosis Detection Kit; US Everbright Inc., China, T6014). Nuclei were stained with DAPI.

### Enzyme-linked immunosorbent assay

The levels (*n* = 9) of inflammatory factors IL-6, IL-1β, and TNF-α in the hippocampus tissue were detected by mouse ELISA. Mice hippocampi were separated after mice were sacrificed according to the manufacturer’s protocol, and hippocampal tissue homogenates were prepared using PBS containing protease inhibitors. Then, the supernatant was collected by centrifugation for 20 min at 4°C, 12,000 rpm, and the protein of the tissue supernatant was quantified by BCA Protein Assay Kit. The diluted solution contained in the ELISA kit was used to treat the samples and quantified to a uniform concentration. Then, the ELISA experiment was carried out according to the manufacturer’s protocol and the absorbance at a 450-nm wavelength was measured by an enzyme-labeled detector. The absorbance of the standard was used to draw a four-parameter logistic curve to calculate the concentration of inflammatory factor in the sample.

### Statistical analysis

All behavioral images and data were monitored and captured by SMART V3.0 behavioral video analysis system. The immunofluorescence staining positive cells were quantified using Image J program. GraphPad Prism software (GraphPad Prism 8) was used for statistical tests, and images were processed using Adobe Photoshop CS6. Student’s *t-*test was used for two-group comparisons, while one-way ANOVA was performed for multiple-group comparisons. All data are shown as mean ± SD.

## Results

### Nuclear localization of α-Syn leads to Ser129 phosphorylation of α-Syn and α-Syn aggregation in the nuclei of mouse hippocampal neurons

**Figure 1:**
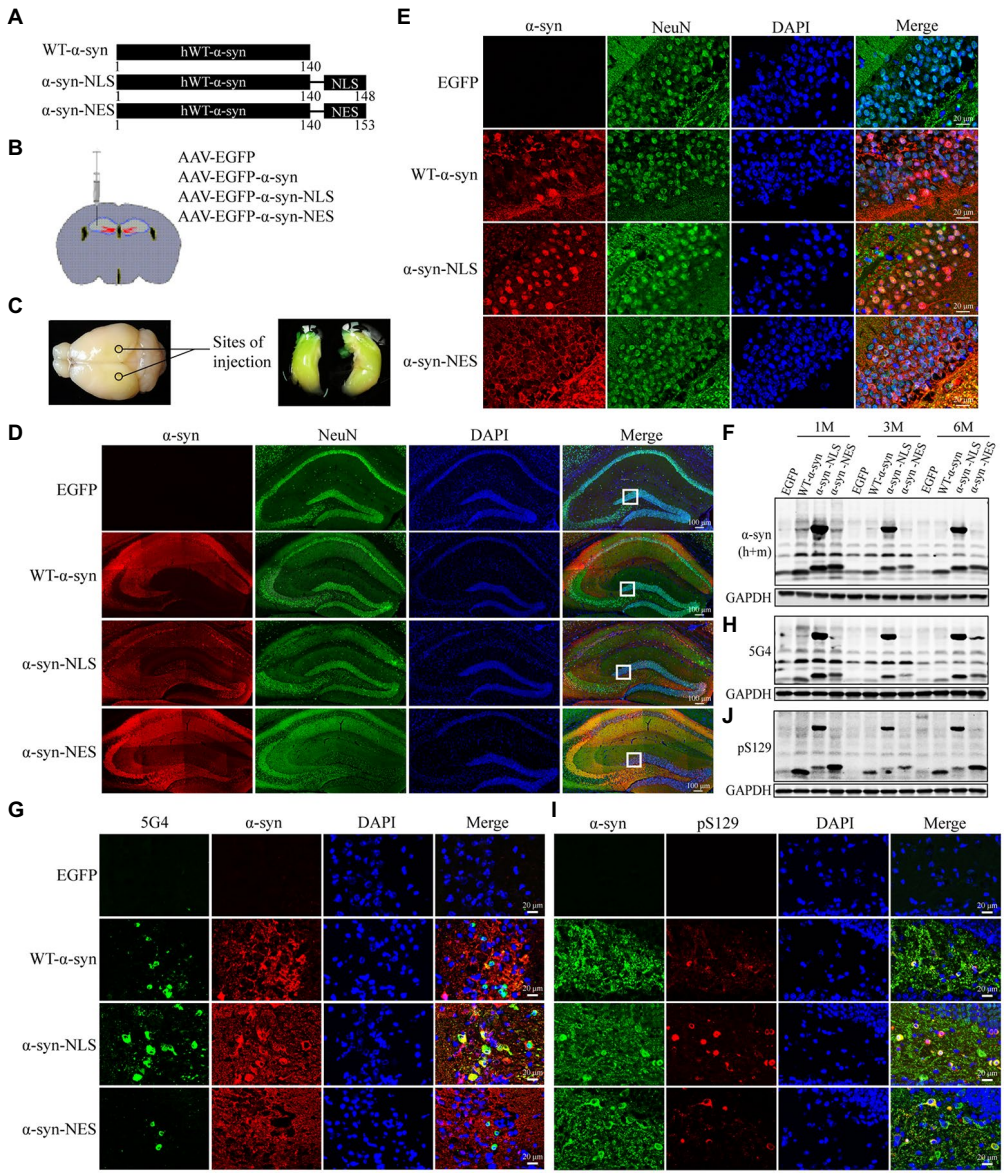
AAV carrying *EGFP-SNCA*-NLS was successfully expressed in the mouse hippocampus and caused significant accumulation of pathological α-syn. **(A)** Plasmids encoding for WT-α-syn, α-syn-NLS, and α-syn-NES were used as template to generate identical constructs to create rAAV2/9. **(B)** Injection sites in the hippocampal region of 6–8-weeks-old mice. **(C)** The virus spread in the hippocampal region of mice after injection. **(D)** Double fluorescent staining of mouse hippocampus with α-syn (red) and neuronal nuclear antigen (NeuN, green) antibodies; DAPI was used to stain all nuclei (blue). **(E)** Immunofluorescence staining reveals subcellular localization of α-syn in the hippocampus. **(F,H,J)** Level of total α-syn, α-syn aggregates (5G4) and Ser129 phosphorylation of α-syn (pS129) expression in mouse models was assessed by western blotting. **(G)** Immunofluorescence image of α-syn aggregates in the hippocampus. **(I)** Immunofluorescence image of Ser129 phosphorylation of α-syn in the hippocampus.

The mouse models of α-syn with specific subcellular expression in the hippocampus were established by injecting rAAV2/9 carrying *EGFP*, *EGFP-SNCA*, *EGFP-SNCA*-NLS, and *EGFP-SNCA*-NES into the hippocampus of mice, where NLS is the nuclear localization signal sequence and NES is the nuclear export signal sequence (Figure 1A). The injection sites are shown in Figures 1B,C. One month later, the expression of α-syn was detected by immunofluorescence staining in the hippocampal specimens. We showed that adeno-associated virus carrying SNCA sequence was successfully expressed in the hippocampus (Figures 1D,F). In α-syn-NLS group and α-syn-NES group, α-syn was located in the nucleus and cytoplasm, respectively, while in WT-α-syn group, α-syn was localized in both cytoplasm and nucleus (Figure 1E). This finding indicated that the mouse models of specific expression of α-syn in different subcellular locations in the hippocampus were successfully established.

Previous studies have shown that about 90% of α-syn deposited in Lewy bodies in the brain of PD patients is phosphorylated at serine-129 (Ser129; Fujiwara et al., 2002; Anderson et al., 2006), suggesting that post-translational modification of α-syn, especially phosphorylation, is related to the pathogenesis of PD. Our immunofluorescence and western blot results showed obvious pathological α-syn accumulation in hippocampal neurons of WT-α-syn, α-syn-NLS, and α-syn-NES groups (Figures 1G–J). Notably, in α-syn-NLS group, in addition to the α-syn monomer at 14 kDa, there was also a part of the 58 kDa tetramer (Figure 1F). A part of α-syn aggregates also appeared in the form of high molecular weight (Figure 1H), and the Ser129 phosphorylation of α-syn also mainly existed in the form of high molecular weight (Figure 1J).

### Nuclear localization of α-Syn leads to motor impairment and memory impairment in mice

**Figure 2:**
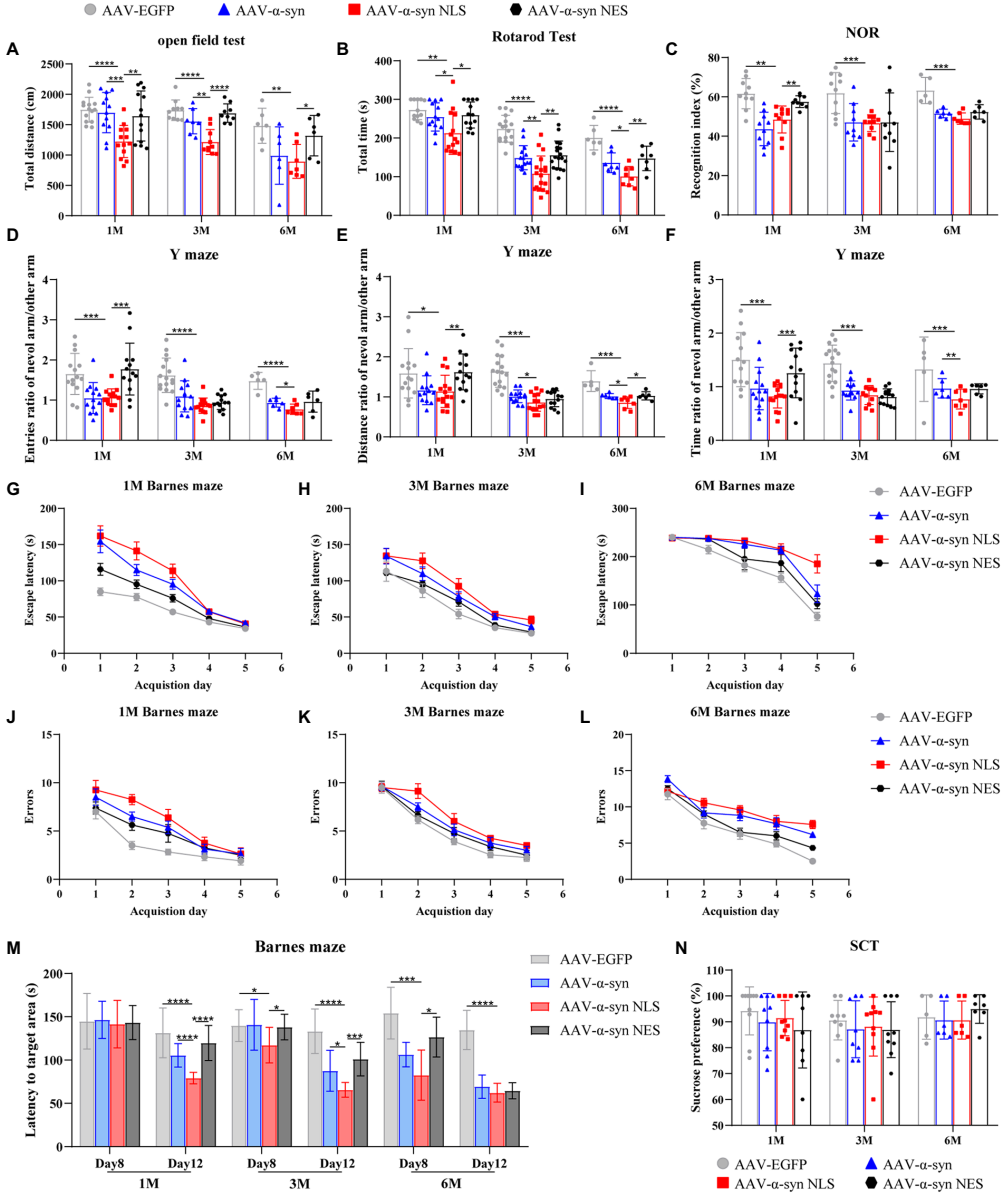
Overexpression of α-syn in hippocampal neuron nuclei causes motor impairment and spatial memory impairment in mice. **(A)** Statistical graph of horizontal total distance in the open-field test (*n* = 15). **(B)** Statistical graph of duration in the rotarod test (*n* = 15). **(C)** Statistical graph of recognition index in novel-object recognition (NOR; *n* = 15). **(D–F)** Statistical graph of the ratio of entries, distance, and time between the novel arm and other arms in the Y maze experiment (*n* = 15). **(G–I)** Statistical graphs of escape time of mice entering the target box from the Barnes maze platform at different detection periods (*n* = 12). **(J–L)** Statistical graphs of error times of mice before entering the target box at different detection periods (*n* = 12). **(M)** Statistical graph of mouse exploration time in the quadrant of the target box after removing the target box from the Barnes maze (*n* = 12). **(N)** Statistical graph of sucrose preference of the sucrose consumption test (SCT; *n* = 10). *p < 0.05, **p < 0.01, ***p < 0.001, ****p < 0.0001.

Next, we used the established mouse model to evaluate the effects of nuclear localization of α-syn on spatial learning and memory ability, motor ability, and mood of the mice. One month, 3 months, and 6 months after the injection of adeno-associated virus, the motor ability of the mice was tested by the open-field test and the rotarod test; the spatial learning and memory ability was tested by the Y maze, novel-object recognition, and Barnes maze; and the depression-like behavior was tested by the sucrose consumption test. The results of the open-field test (Figure 2A) and the rotarod test (Figure 2B) showed that, compared with the control group, the mice in α-syn-NLS group showed motor impairment at month 1, and this deficit persisted throughout the experiment. WT-α-syn and α-syn-NES groups also showed motor impairment, but it was not as serious as that in α-syn-NLS group. The results of novel-object recognition (Figure 2C) and Y maze test (Figures 2D–F) showed that the short-term memory ability of WT-α-syn and α-syn-NLS mice was impaired at month 1, while α-syn-NES mice did not show obvious memory defects at month 1. Compared with the control group, the mice in the other three groups showed obvious short-term memory impairment at months 3 and 6. The results of the Barnes maze (Figures 2G–M) in three time periods showed that the mice in α-syn-NLS group needed longer escape time and forgot more earlier. We showed that nuclear localization of α-syn was able to more rapidly cause significant deficits in spatial learning and long-term memory storage in mice, but there was no significant effect on sucrose preference (Figure 2N).

Taken together, the results of the behavioral tests showed that compared with α-syn overexpression group and α-syn nuclear export group, nuclear localization of α-syn could lead to earlier motor impairment and memory impairment in mice, but would not cause depression-like behavior.

### Nuclear localization of α-Syn can cause DNA damage in hippocampal neurons of mice

**Figure 3:**
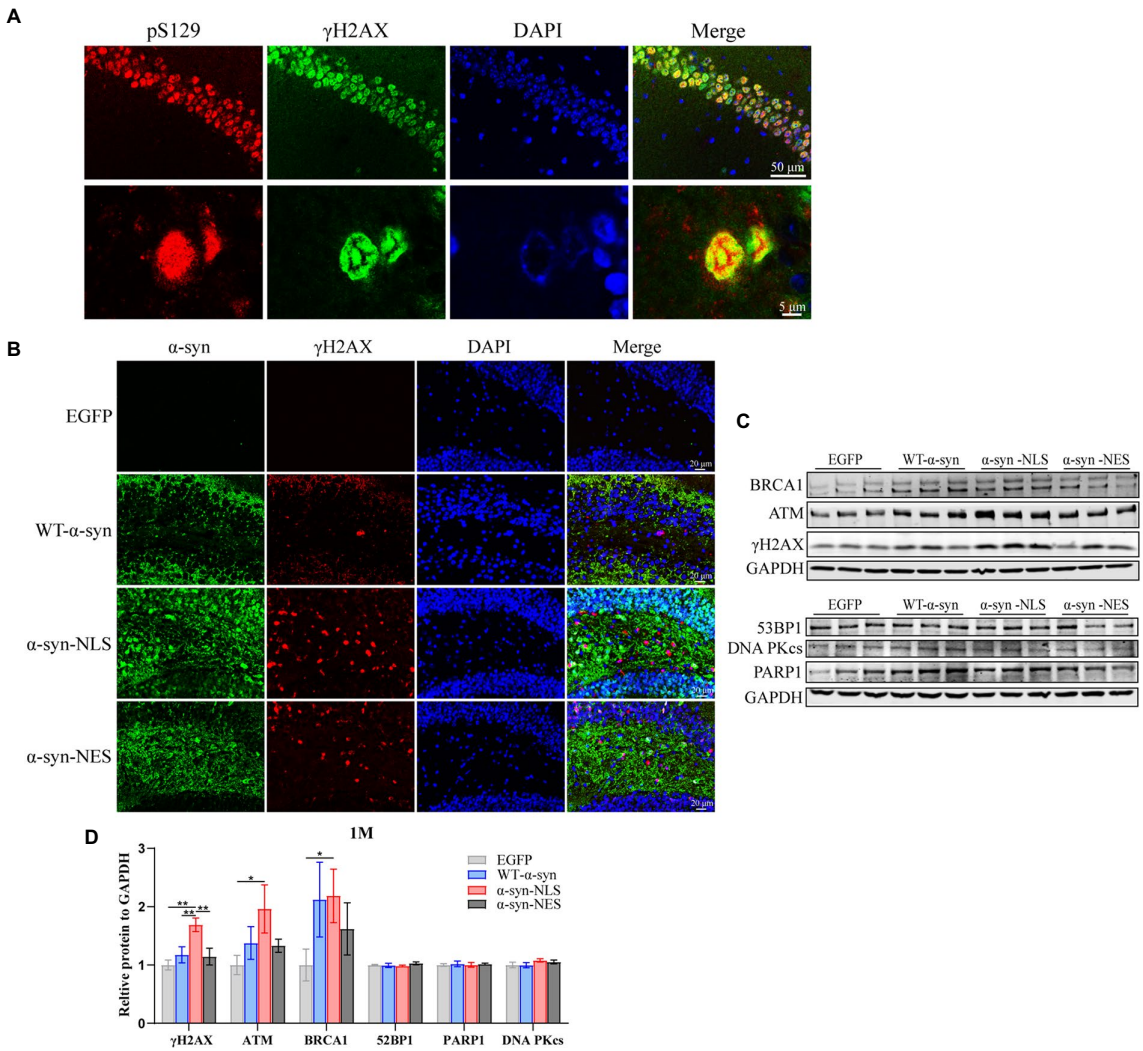
Nuclear localized α-syn induces DNA damage in mouse hippocampal neurons. **(A)** Confocal microscopy scans of double fluorescent staining of hippocampal tissue sections in the α-syn-NLS group at month 1 with antibodies to pS129 (red) and γH2AX (green). **(B)** Immunofluorescence staining of mouse hippocampus with α-syn (green) and γH2AX (red) antibodies after 1 month of virus injection; DAPI was used to stain all nuclei (blue). **(C)** Western blot of the hippocampus tissues of mice with antibodies to γH2AX, ATM, BRCA1, 53BP1, PARP1, and DNA PKcs (*n* = 3). **(D)** Relative levels of proteins (ratio to GAPDH) on western blot are presented (*n* = 3). *p < 0.05, **p < 0.01.

Previous studies have shown that overexpression of α-syn in nuclei can lead to downregulation of genes related to DNA damage repair (Paiva et al., 2017), thereby causing DNA damage response. Our results showed that α-syn localized in the nucleus promoted the phosphorylation level of Ser129. Therefore, we used both pS129 antibody and serine-139 phosphorylated histone γH2AX antibody to double fluorescent stain the hippocampus slices of α-syn-NLS mice 1 month after virus injection, and confocal microscope scanning showed that they were co-localized (Figure 3A). The western blot results showed that nuclear localization of α-syn caused upregulation of telangiectatic ataxia mutant (ATM) and downstream γH2AX and BRCA1 (Figures 3C,D), where γH2AX was the main marker of DNA damage, and the immunofluorescence results showed the same pattern (Figure 3B). These results indicated that nuclear localization of α-syn could increase the DNA damage reaction of hippocampal neurons in mice.

### DNA damage of hippocampal neurons may activate abnormal neuronal cell cycle

**Figure 4:**
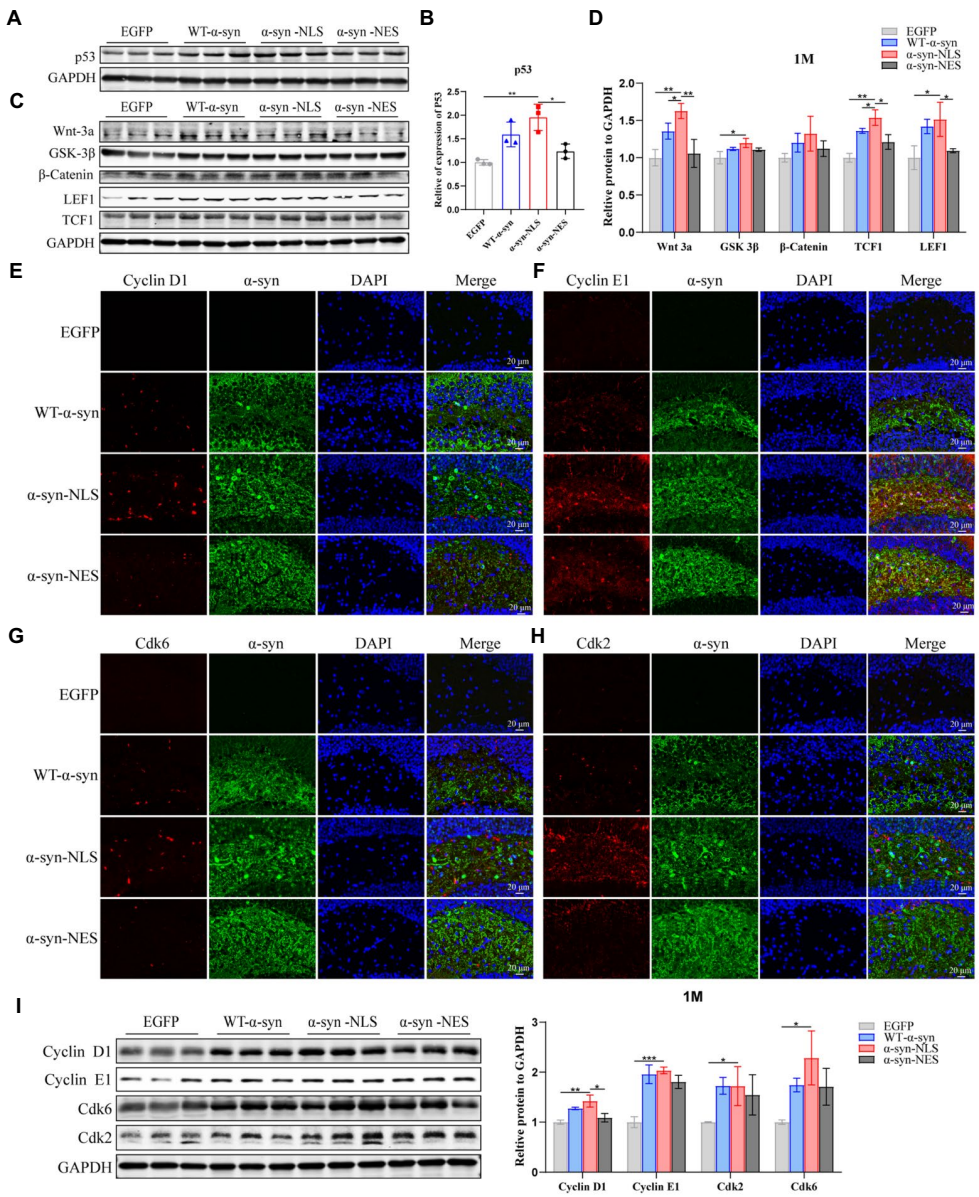
DNA damage activates cell cycle G1/S checkpoints, prompting neuronal cell cycle reentry. **(A,B)** Western blot and relative level analysis of p53 protein after 1 month of virus injection (*n* = 3). **(C)** Western blot of the hippocampus tissues of mice with antibodies to Wnt-3a, glycogen synthase kinase-3beta (GSK-3β), β-Catenin, LEF1, and TCF1 (*n* = 3). **(D)** Relative levels of proteins (ratio to GAPDH) in western blot are presented (*n* = 3). **(E–H)** The expression levels of cell cycle S phase proteins CDK2, CDK6, Cyclin D1, and Cyclin E1 were detected by immunofluorescence. **(I)** Western blot with CDK2, CDK6, Cyclin D1, and Cyclin E1 antibodies and their relative levels (*n* = 3). *p < 0.05, **p < 0.01, ***p < 0.001.

Recent studies have suggested that terminally differentiated cells, including neurons, can actually repair DNA damage (Kruman, 2004). Their repair process includes the core of cell cycle mechanism and a series of monitoring channels called cell cycle regulatory points. When the cell’s DNA is damaged, p53 gene can be used as a transcription factor to start the expression of CDKN1A, and then block the cell cycle in G1 phase, which can obtain time for DNA damage repair before the cell enters S phase (Kruman, 2004; Chen et al., 2007; Mattia et al., 2007). Our results showed that p53 was significantly upregulated in α-syn-NLS group 1 month after virus injection (Figures 4A,B). Studies have shown that α-syn can activate Wnt/β-catenin signaling pathway (Paiva et al., 2017). Wnt signaling pathway helps neural stem cell proliferation by increasing the number of cells in S phase and shortening the time in G1 phase, and catenin plays an important role in promoting the proliferation and self-renewal of neural stem cells (Chenn and Walsh, 2002). We found that overexpression of α-syn induced upregulation of proteins related to Wnt signaling pathway, and α-syn-NLS group showed the most obvious upregulation (Figures 4C,D), indicating that nuclear localization of α-syn was more likely to activate the Wnt signaling pathway, change cell cycle, and promote cell division. Therefore, we used immunofluorescence and western blot to detect the expression levels of cyclin kinases CDK2 and CDK6, and cyclin Cyclin D1 and Cyclin E1. The results showed that the expression levels of these four proteins were all upregulated in α-syn-NLS group (Figures 4E–I), indicating that the G1 phase of neural precursor cells shortened and that the cells entered the S phase.

**Figure 5:**
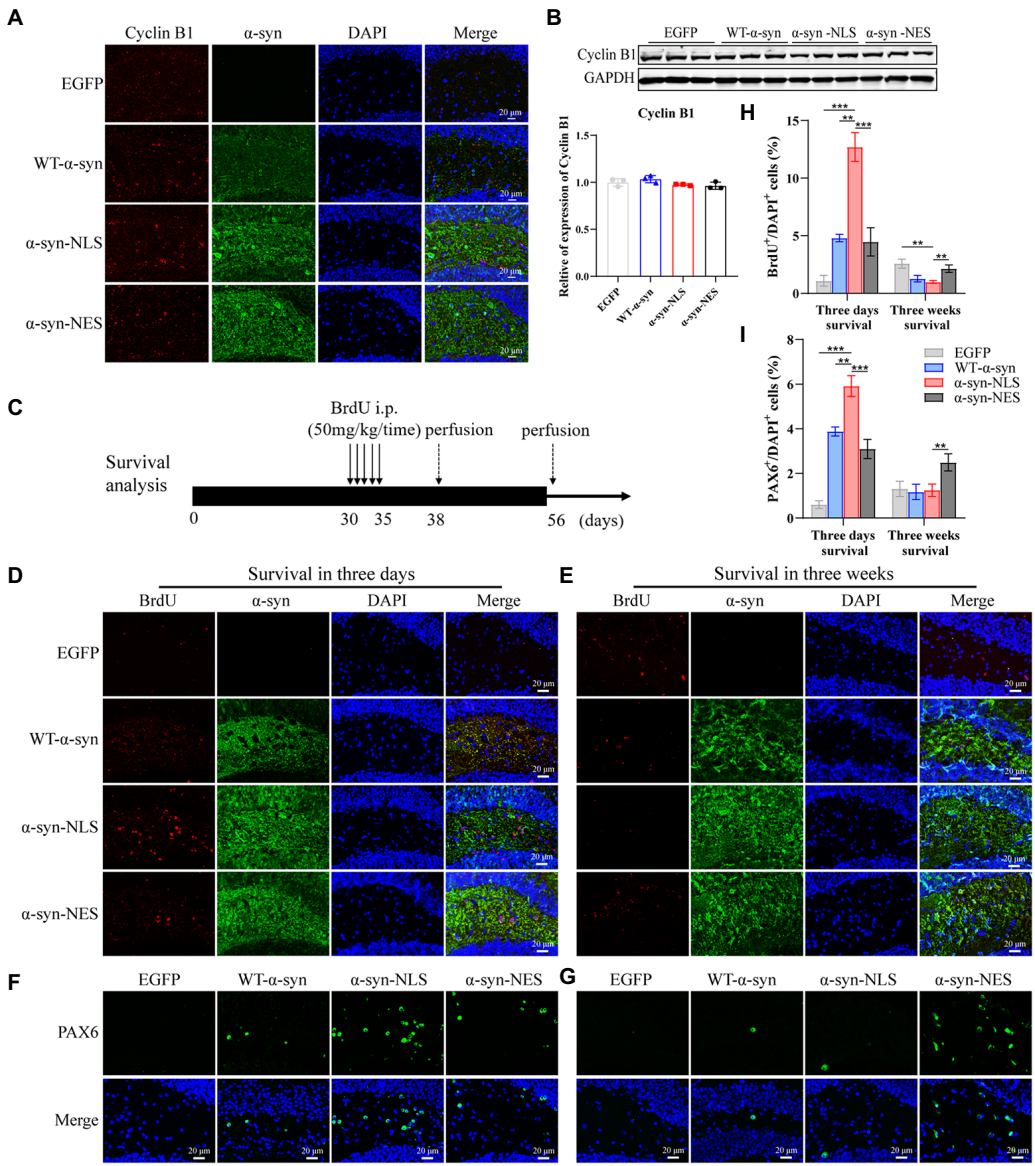
Cell cycle progression is blocked before G2 phase and cells exit the cell cycle. **(A)** The expression of cell cycle G2 phase protein Cyclin B1 was detected by immunofluorescence. **(B)** Western blot and relative level analysis of Cyclin B1 protein after 1 month of virus injection (*n* = 3). **(C)** A pattern diagram for detecting neuronal cell survival. BrdU was injected once a day starting from month 1 after virus injection for five consecutive days. BrdU-positive cells were examined on day 3 (survival 3-days) and day 21 (survival 3-weeks) after last BrdU injection. **(D)** Representative BrdU-positive cells on day 3. **(E)** Representative BrdU-positive cells on day 21. **(F)** Representative PAX6-positive cells on day 3. **(G)** Representative PAX6-positive cells on day 21. **(H)** BrdU^+^ cells were quantified of three-days survival and three-weeks survival (*n* = 4). **(I)** PAX6^+^ cells were quantified of three-days survival and three-weeks survival (*n* = 4). **p < 0.01, ***p < 0.001.

Previous studies have shown that, although under the condition of apoptosis, neurons can enter the S phase and DNA replication occurs, the last stage of cell cycle is not observed in dying neurons (Greene et al., 2004). We detected the expression level of Cyclin B1 in G2 phase by immunofluorescence and western blot, and the results showed that there was no significant difference in protein expression of Cyclin B1 at month 1 (Figures 5A,B), which indicated that the neuron cell cycle was blocked before G2 phase, and that the cells withdrew from the cell cycle process.

In order to explore whether this disordered cell cycle would affect the survival and differentiation of cells, we injected BrdU intraperitoneally into the mice and detected it through immunofluorescence. BrdU, as a thymidine analog, can replace thymidine in the DNA synthesis process and be incorporated into the newly synthesized DNA (Taylor et al., 1957), so it can be used to detect the proliferation, differentiation, and migration of neuronal cells. Figure 5C shows a pattern diagram for detecting the survival rate of neuron cells. One month after virus injection, BrdU was injected continuously for 5 days, and BrdU-positive cells were detected on the third day (survival 3 days) and 21st day (survival 3 weeks) after the last BrdU injection. The results showed that the number of BrdU-positive cells in α-syn-NLS group increased significantly at the three-day survival time (Figures 5D,H), and the number of PAX6-positive cells, which represented the shortening of G1 phase of neural precursor cells, also increased (Figures 5F,I). Three weeks after the last injection of BrdU, most BrdU-labeled positive cells in α-syn-NLS group died (Figures 5E,H), and the number of PAX6-positive cells showed the same trend (Figures 5G,I), indicating that the nerve cells that entered the S phase of cell cycle could not completely proliferate, and then were cleared by the body.

### DNA damage in the hippocampus caused by nuclear localization of α-Syn increases with time in mice

**Figure 6:**
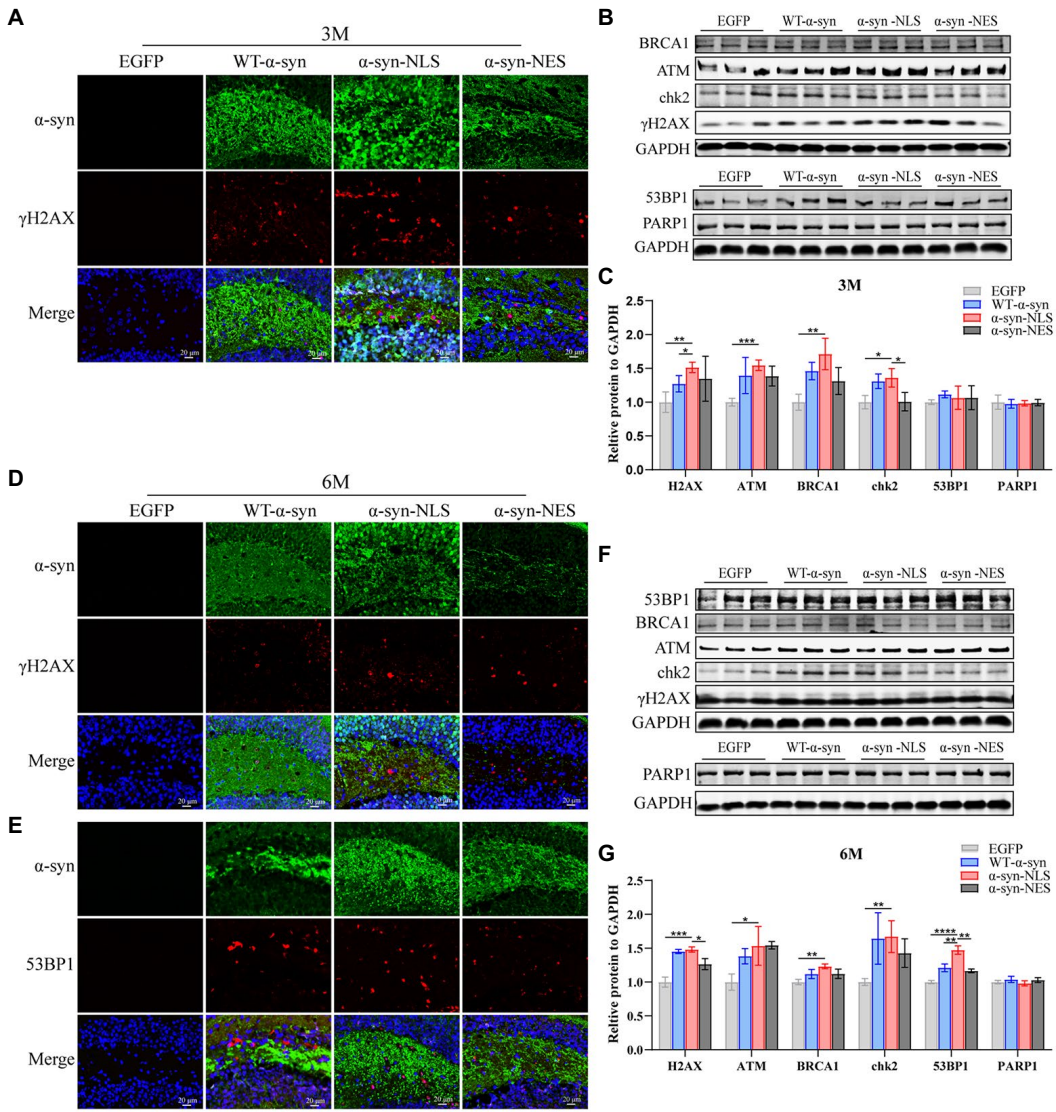
DNA damage caused by nuclear localized α-syn aggravates over time. **(A,D)** Immunofluorescence staining of mouse hippocampus with α-syn (green) and γH2AX (red) antibodies at months 3 and 6 after virus injection; DAPI was used to stain all nuclei (blue). **(B,F)** Western blot of the hippocampus tissues of mice with antibodies to γH2AX, ATM, BRCA1, chk2, 53BP1, and PARP1 (*n* = 3). **(C,G)** Relative levels of proteins (ratio to GAPDH) in western blot are presented (n = 3). **(E)** Immunofluorescence staining of mouse hippocampus with α-syn (green) and 53BP1 (red) antibodies at month 6 after virus injection; DAPI was used to stain all nuclei (blue). *p < 0.05, **p < 0.01, ***p < 0.001, ****p < 0.0001.

Our results showed that the localization of α-syn at month 1 after virus injection led to DNA damage (Figures 3B,C), and similar results were found at months 3 and 6 post virus injection. The nuclear localization of α-syn led to upregulation of γH2AX, ATM, and downstream proteins (Figures 6A–D,F,G). Moreover, in the 6-month test, we were found that p53-binding protein 1 (53BP1), which showed no significant difference in expression in the first two time periods, was also upregulated (Figures 6E–G).

**Figure 7:**
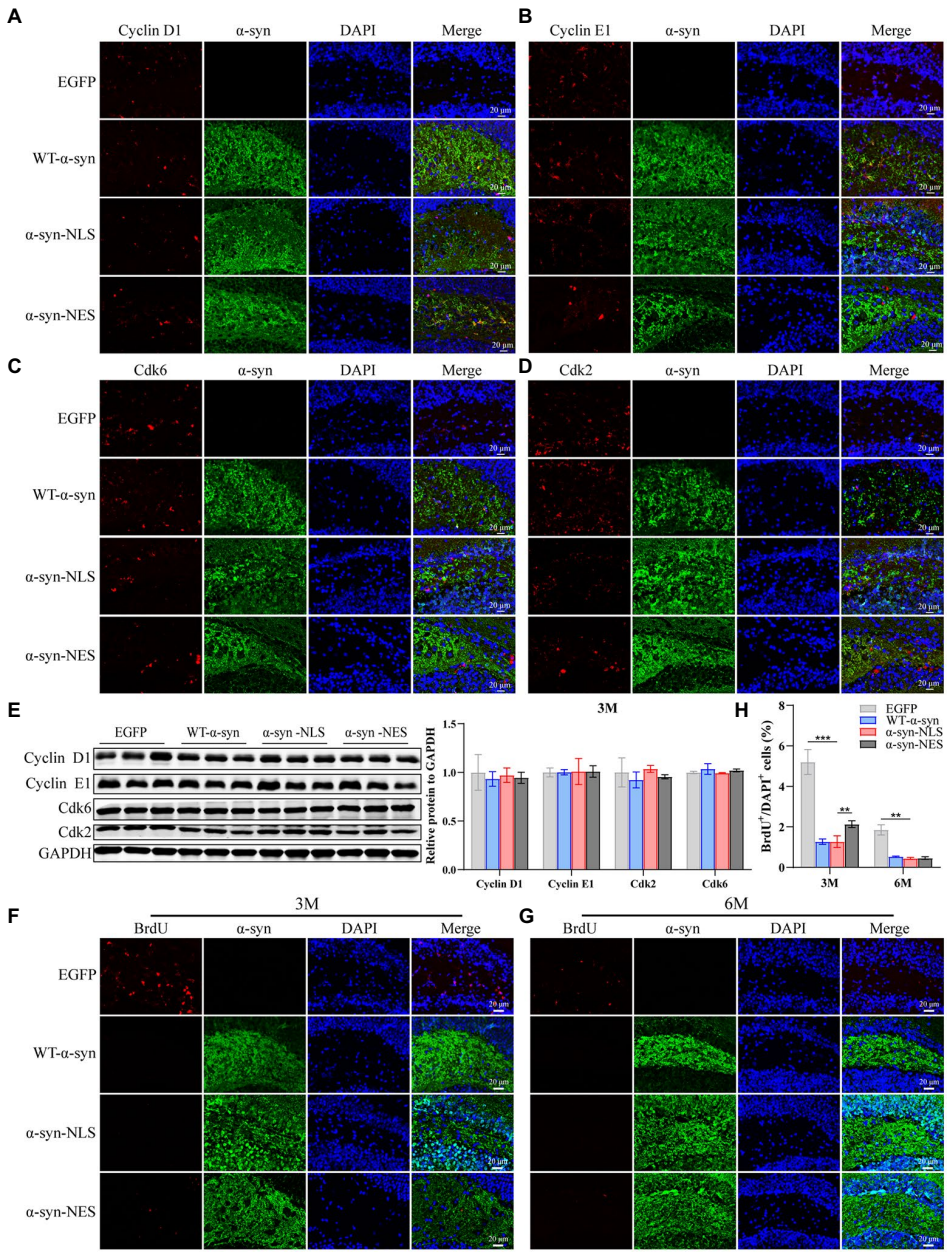
Long-term overexpression of α-syn inhibits neural cell proliferation. **(A–D)** The expression of cell cycle S phase proteins CDK2, CDK6, Cyclin D1, and Cyclin E1 was detected by immunofluorescence at month 3 after virus injection. **(E)** Western blot with CDK2, CDK6, Cyclin D1, and Cyclin E1 antibodies at month 3 after virus injection and their relative levels (*n* = 3). **(F)** Representative BrdU-positive cells staining image at month 3 after virus injection. **(G)** Representative BrdU-positive cells staining image at month 6 after virus injection. **(H)** BrdU^+^ cells were quantified at months 3 and 6 after virus injection (*n* = 4). **p < 0.01, ***p < 0.001.

Then, we re-examined the expression levels of S phase proteins CDK2, CDK6, Cyclin D1, and Cyclin E1 at month 3 after virus injection. The results showed that there was almost no difference in the expression of S phase proteins among several groups at month 3 (Figures 7A–E), suggesting that nerve cells did not reactivate and re-enter the cell cycle at month 3. In addition, compared with the control mice, the immunofluorescence detection results at months 3 and 6 showed that the number of newly proliferated cells in WT-α-syn, α-syn-NLS, and α-syn-NES groups decreased (Figures 7F–H), indicating that long-term overexpression of α-syn could inhibit the proliferation of nerve cells.

### Nuclear localization of α-Syn can lead to apoptosis of neurons in mouse hippocampus

**Figure 8:**
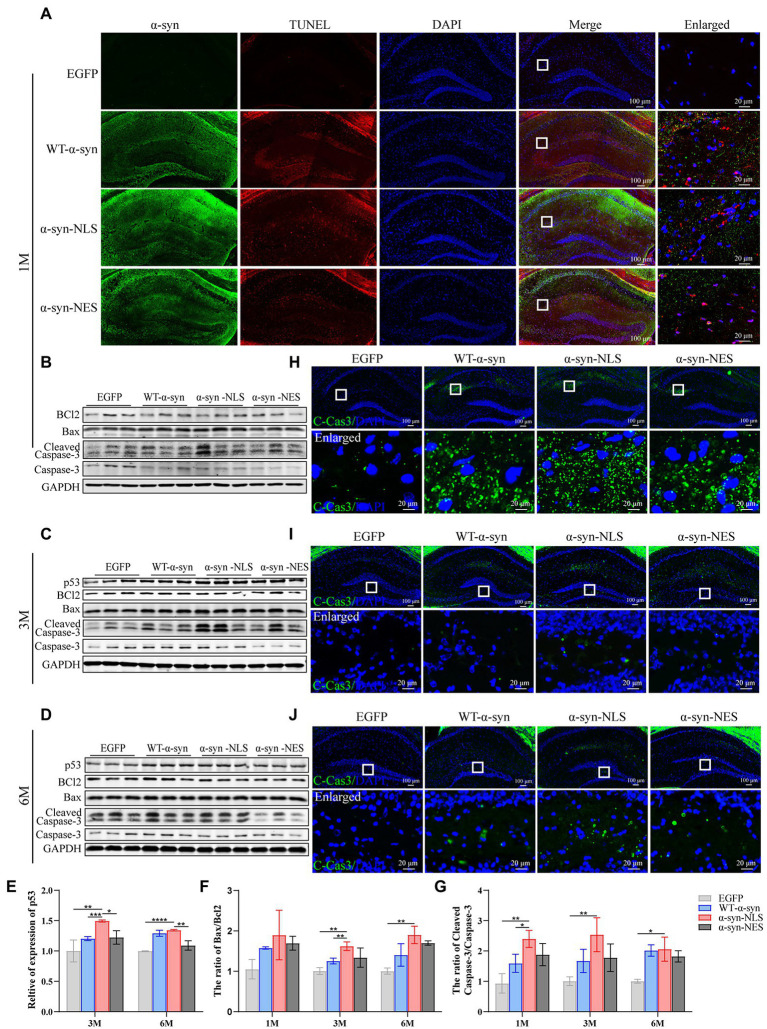
Nuclear localized α-syn causes neuronal apoptosis in the mouse hippocampus. **(A)** TUNEL staining in the hippocampus at month 1 after virus injection. **(B–D)** Western blot of mouse hippocampus at months 1, 3, and 6 after virus injection with p53, BCl2, Bax, Cleaved Caspase 3 (C-Cas3), and Caspase 3 antibodies (*n* = 3). **(E–G)** Relative levels of proteins (ratio to GAPDH) in western blot (*n* = 3). **(H–J)** Immunofluorescence staining of mouse hippocampus with C-Cas3 (green) antibody at months 1, 3, and 6 after virus injection; DAPI was used to stain all nuclei (blue). *p < 0.05, **p < 0.01, ***p < 0.001, ****p < 0.0001.

Previous studies have shown that neuronal apoptosis occurs when neuron’s attempt to enter the cell cycle is aborted (Samuel et al., 2002). There are some common factors in the process of DNA damage repair, apoptosis, and cell cycle regulation, such as p53, and it has been shown that some apoptosis induced by DNA damage is p53-dependent (Kruman, 2004; Becker and Bonni, 2005). Our western blot results showed that the expression of apoptosis-related proteins Bax and Cleaved-Caspase3 (C-Cas3) was observed in WT-α-syn, α-syn-NLS, and α-syn-NES groups when p53 protein was upregulated (Figures 8B–G), and the upregulation of apoptosis-related proteins in α-syn-NLS group was more significant than that in the other two groups. TUNEL staining (Figure 8A) and C-Cas3 immunofluorescence staining (Figures 8H–J) results showed the presence of apoptosis in the hippocampus of WT-α-syn group, α-syn-NLS group, and α-syn-NES group. The immunofluorescence results at month 1 after injection showed that apoptosis mainly occurred in CA1 and CA2 regions of the hippocampus (Figures 8A,H). However, at month 3, the expression of C-Cas3 first appeared in the polymorphic layer of hippocampal dentate gyrus in α-syn-NLS group (Figure 8I). At month 6, the polymorphic layer of the dentate gyrus in WT-α-syn, α-syn-NLS, and α-syn-NES groups showed the staining results of C-Cas3 (Figure 8J), and the α-syn-NLS group was the most obvious. Therefore, nuclear localization of α-syn induced obvious apoptosis in the hippocampus, and there was a trend of increasing the number of apoptotic cells with time.

### Nuclear localization of α-Syn can induce a more serious inflammatory reaction in mouse hippocampus

**Figure 9:**
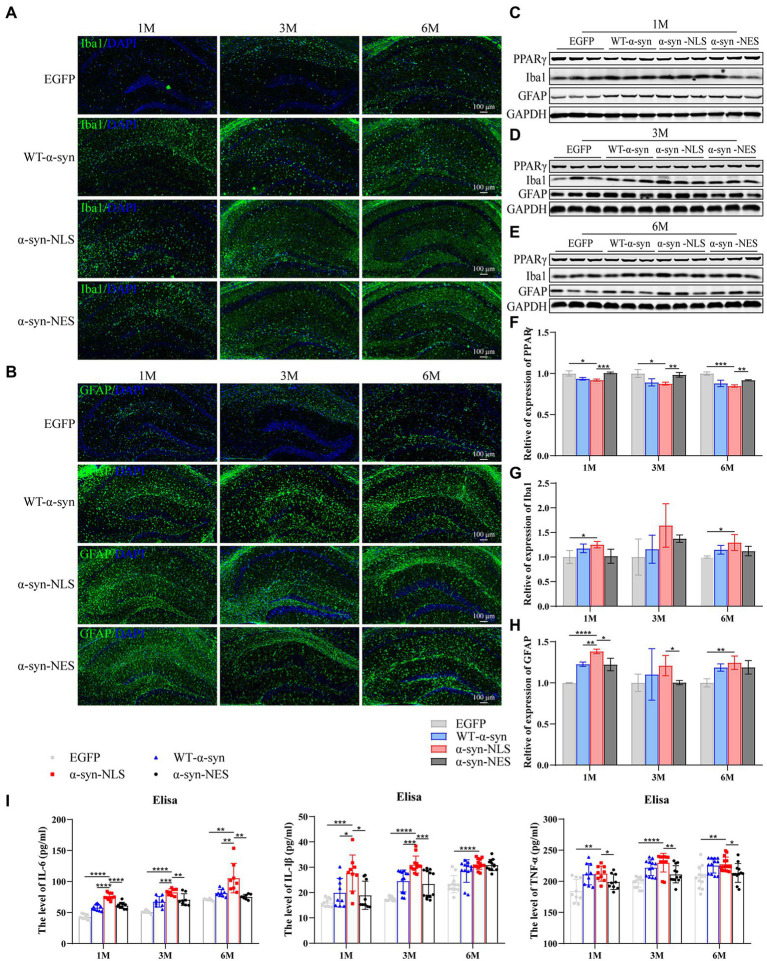
Nuclear localization α-syn increase astrogliosis in hippocampus and induces inflammatory response. **(A)** Immunofluorescence staining of mouse hippocampus with Iba1 (green) antibody at months 1, 3, and 6 after virus injection; DAPI was used to stain all nuclei (blue). **(B)** Immunofluorescence staining of mouse hippocampus with GFAP (green) antibody at months 1, 3, and 6 after virus injection; DAPI was used to stain all nuclei (blue). **(C–E)** Western blot of mouse hippocampus at months 1, 3, and 6 after virus injection with PPARγ, Iba1, and GFAP antibodies (*n* = 3). **(F)** Level of PPARγ expression was assessed by western blot (*n* = 3). **(G)** Level of Iba1 expression was assessed by western blot (*n* = 3). **(H)** Level of GFAP expression was assessed by western blot (*n* = 3). **(I)** The level of IL-6, IL-1β, and TNF-α in the hippocampus by Elisa (*n* = 9). *p < 0.05, **p < 0.01, ***p < 0.001, ****p < 0.0001.

As resident macrophages in the brain, microglia are particularly sensitive to the cell damage signals generated by dysfunctional neurons, which leads to their activation and phagocytosis and removal of the damaged fragments (Milner and Campbell, 2003). The activation of microglia is one of the mechanisms of oxidative stress caused by the accumulation of α-syn. Our results showed a significant downregulation of peroxisome proliferator activated receptor γ (PPARγ) in α-syn-NLS group at months 1, 3, and 6 (Figures 9C–F), and PPARγ played an important role in inhibiting microglia activation and neuron loss. The following results also showed that a large number of microglia (Iba1) were activated in WT-α-syn, α-syn-NLS, and α-syn-NES (Figures 9A,C–E,G), and the expression of Iba1 was upregulated most significantly in α-syn-NLS group. Factors derived from microglia, including pro-inflammatory factors, anti-inflammatory factors, and oxidative stress substances, could trigger astrocyte receptors activation, thereby inducing inflammation (Jha et al., 2019). Our results showed that the expression of pro-inflammatory factors IL-6, IL-1β, and TNF-α in the hippocampus of α-syn-NLS group increased significantly (Figure 9I), and at the same time, astrocytes (GFAP) also showed obvious proliferation (Figures 9B–E,H). Therefore, nuclear localization of α-syn could induce a more serious inflammatory reaction in mouse hippocampus.

## Discussion

There is increasing evidence that the pathological accumulation of α-syn in the hippocampus may lead to cognitive impairment (Regensburger et al., 2014; Kasongo et al., 2020; Zhang et al., 2022). Our research showed that compared with other groups, nuclear localization of α-syn caused DNA damage response in hippocampal neuron earlier, induced apoptosis and inflammatory reaction, thereby causing more rapid and serious cognitive impairment and motor impairment in mice.

Previous studies have shown that α-syn exists as a monomer and a tetramer in natural state. Stable tetramer can resist the aggregation of α-syn, but at the same time, the structure of this tetramer is easily affected by physiological environment, and it is more likely to cause wrong folding and aggregation of α-syn in the process of dissociation into monomer, resulting in neurotoxicity and intracellular inclusion body (Bartels et al., 2011; Corbett and Kordower, 2015; Evans et al., 2021). After we injected the adeno-associated virus carrying *EGFP-SNCA*-NLS into the hippocampus of mice, both α-syn monomer and tetramer were present in the hippocampus, and the Ser129 phosphorylation of α-syn mostly existed in the form of high molecular weight. Therefore, we speculated that the nuclear localized α-syn was more likely to fold to form tetramers, but its tetramer structure was unstable; instead, it was more likely to undergo phosphorylation modification, resulting in pathological accumulation of α-syn. And we hypothesized that the phosphorylation of α-syn in high molecular weight might be more likely to trigger the pathological accumulation and toxic effects of α-synuclein.

The phosphorylation of α-syn at Ser129 can promote the accumulation of oligomer α-syn *in vitro* and accelerate the formation of inclusion bodies (Sugeno et al., 2008). Further studies have shown that the hyperphosphorylation of α-syn may affect its solubility, membrane binding characteristics, and subcellular distribution (Zhou et al., 2011; Visanji et al., 2020). We speculated that nuclear localization of α-syn could cause Ser129 phosphorylation of α-syn in the nucleus, and the phosphorylated α-syn further would promote the deposition and aggregation of α-syn. In addition, α-syn entering the nucleus probably interacted with DNA, which changed the physical and chemical properties of DNA. In turn, DNA may also change the spatial conformation of α-syn (Hegde and Rao, 2007; Hegde et al., 2010), leading to the pathological accumulation of α-syn. This was verified by double fluorescence staining with pS129 antibody and γH2AX antibody. These suggest that the relationship between α-syn and DNA repair in neurons may be an intimate one, with many potentially interesting links.

It has been shown that both WT-α-syn and A30P α-syn could promote the downregulation of genes involved in DNA repair and the increase of DNA damage response (Paiva et al., 2017). Double-strand breaks (DSBs) are the most serious type of DNA damage. After DSBs are formed, the breakpoint of histone H2AX at serine-139 is rapidly phosphorylated to form γH2AX, which participates in the DNA damage response process (Marková et al., 2007; Löbrich et al., 2010). Then, γH2AX acts as a docking station for other DNA damage signal molecules (such as 53BP1), which accumulate in a histone modification-dependent manner to form lesions, mediate cell cycle to repair damaged DNA, or induce apoptosis to clear cells that are hopeless for repair (Ciccia and Elledge, 2010; Panier and Boulton, 2014). We found that the injection of nuclear localization α-syn in mice significantly increased γH2AX after 1 month, and the number of hippocampal neurons in the S phase of cell cycle increased, which may be an attempt of hippocampal neuronal cells to repair DNA damage. However, the research on Alzheimer disease has suggested that after the differentiated neurons re-enter the cell cycle, they cannot complete the M phase, and finally undergo apoptosis (Husseman et al., 2000; Bonda et al., 2010). Our results have also reached a similar conclusion. The number of G2 cells did not change significantly after 1 month of nuclear localization of α-syn, and there were a large number of apoptotic cells. This indicates that the re-entry of cell cycle could not be carried out completely, and that the nerve cells with disordered cell cycle have to eliminated through apoptosis pathway. During the whole experiment, the high expression of ATM and γH2AX always existed in α-syn-NLS group, and the 53BP1 protein, which had no obvious difference during the first two periods, also showed up-regulation at month 6. It could interact with BRCA1 and other DNA damage sensors, and regulate the functions of cell apoptosis and cell cycle arrest to determine the fate of cells (Mirza-Aghazadeh-Attari et al., 2019), meaning that the DNA damage reaction persisted and gradually worsened with time. We speculate that excessive or abnormal DNA repair may promote the formation of α-syn aggregates, which enter the cytoplasm or could be transported to other cells, further promoting the Lewy pathology propagation from cell-to-cell, resulting in the persistence of DNA damage reaction. We failed to detect the increase in S phase cells after 3 months of nuclear localization of α-syn, accompanied by the decrease of newly proliferating cells and the increase of apoptotic cells, which indicated that after 3 months, the cell cycle was no longer activated for DNA damage repair, and the cells hopeless for repair were cleared through the apoptotic pathway.

Studies have shown that α-syn accumulation can act as damaged-associated molecular patterns (DAMPs) to trigger the immune system and neuroinflammatory processes, and α-syn can interact directly with microglia to induce pro-inflammatory signaling and inflammatory response (Cao et al., 2012; Kim et al., 2013). Our results showed that the nuclear localization of α-syn always caused the upregulation of oxidative stress level and the increase of pro-inflammatory factors release. At the same time, the cell damage signal promoted the activation of microglia and astrocytes, which further promoted the strengthening of inflammatory response. Prior studies have shown that one of the effects of neuroinflammation is to reduce neurogenesis (Gebara et al., 2016), and excessive pro-inflammatory factors could increase the quiescence of neural stem cells and promote the failure of stem cell pool. Therefore, we suspected that the increase of inflammatory reaction in mice might directly affect the activation state of neural stem cells and play a role in inhibiting the number of neural stem cells, thereby affecting the learning and storage of new memories in mice. Furthermore, the increase of neuronal apoptosis and the strengthening of inflammatory reaction might increase the vulnerability of mouse cells to DNA damage, weaken their repair function, and promote the increase of DNA damage reaction, and then the number of apoptotic cells would continue to increase, which would further lead to the proliferation of glial cells and the decline of neurogenesis. Such a vicious circle would eventually affect the function of the hippocampus, leading to motor impairment and cognitive impairment in mice.

**Figure 10:**
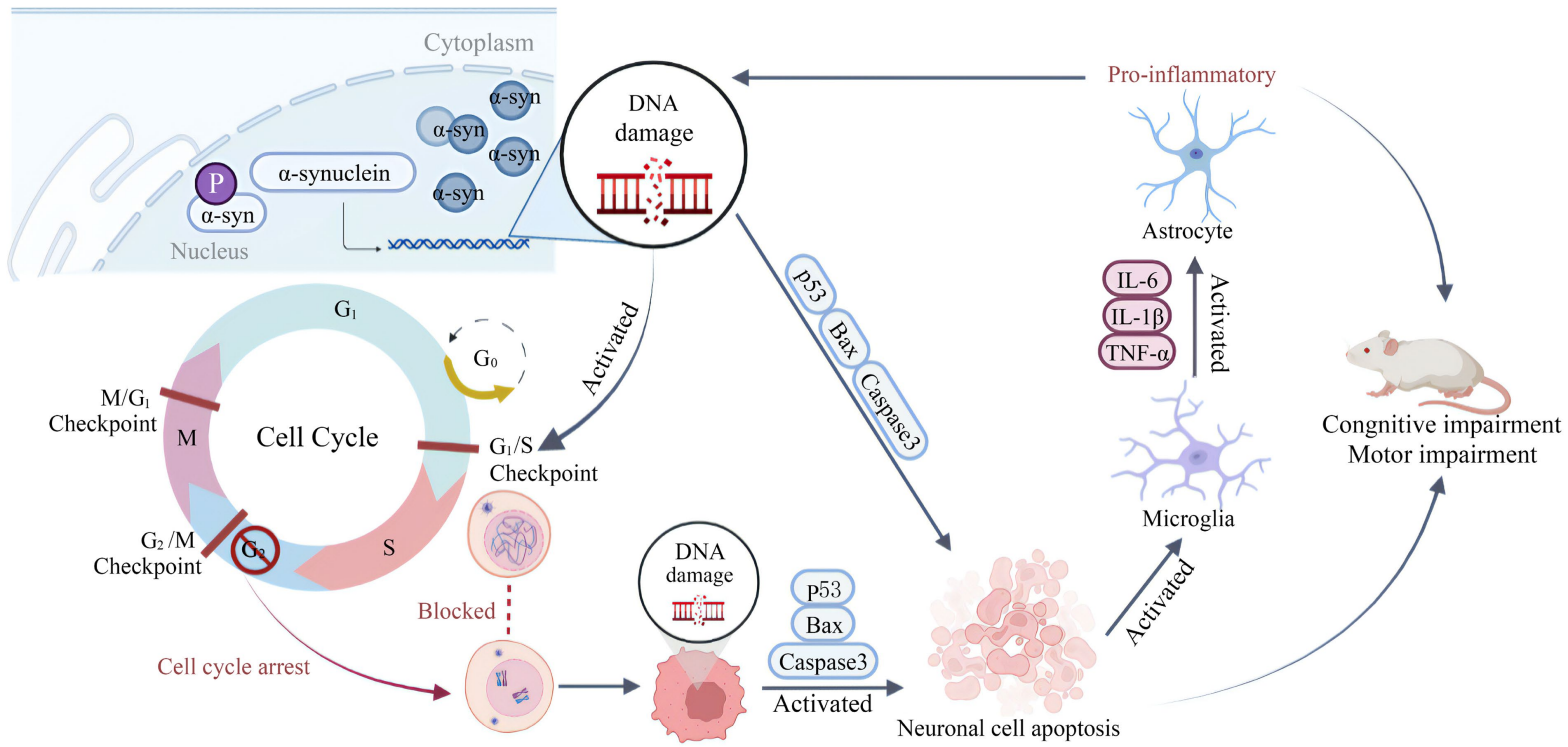
Schematic diagram for summarizing the behavioral impairment in mice caused by overexpression of α-syn in hippocampal neuron nuclei. The nuclear overexpression of α-syn causes DNA damage in hippocampal neurons, and leads to cell cycle activation and abnormal blockade, further induced hippocampal neuronal apoptosis and inflammatory response. Then the inflammation further aggravates DNA damage, creating a vicious cycle that ultimately promotes cognitive impairment and motor impairment in mice. The schematic illustration was drawn according to BioRender (https://biorender.com/).

In a word, our results showed that nuclear localization of α-syn could lead to memory impairment and motor impairment in mice. The specific molecular mechanism might be as shown in Figure 10. The nuclear overexpression of α-syn causes DNA damage reaction of hippocampal neurons, which leads to activation and abnormal blocking of cell cycle, and further induced apoptosis of hippocampal neurons and inflammatory reaction, which in turn further aggravate DNA damage and form a vicious circle. These results suggest that nuclear localized α-syn may directly affect the normal function of hippocampus by aggravating DNA damage, causing apoptosis and inflammation, and triggering PD-related symptoms. However, since there are functional circuits between the hippocampus and cortex, amygdala and other brain regions, the impairment of working memory and motor ability may also involve the impairment of hippocampus and cortex circuits (Burwell et al., 2004; Card et al., 2005). Whether α-syn affects the operation of these circuits needs further research to help us further understand the deposition and diffusion mechanism of α-syn.

## Data availability statement

The raw data supporting the conclusions of this article will be made available by the authors, without undue reservation.

## Ethics statement

The animal study was reviewed and approved by the Experimental Animal Ethic Committee of the Institute of Medical Biology Chinese Academy of Medical Sciences (DWSP202203024).

## Author contributions

YP performed experiments and wrote the manuscript. QZ, GL, TD, ZH, and YZ performed experiments. YP, ZW, and KM designed experiments. ZH, YZ, and KM revised the manuscript. All authors read and approved the final manuscript.

## Funding

This study was supported by the CAMS Innovation Fund for Medical Sciences (CIFMS; NO. 2021-I2M-1-043), the Technology Innovation Talents Project of Yunnan Province (NO. 202105AD160018).

## Conflict of interest

The authors declare that the research was conducted in the absence of any commercial or financial relationships that could be construed as a potential conflict of interest.

## Publisher’s note

All claims expressed in this article are solely those of the authors and do not necessarily represent those of their affiliated organizations, or those of the publisher, the editors and the reviewers. Any product that may be evaluated in this article, or claim that may be made by its manufacturer, is not guaranteed or endorsed by the publisher.

## Abbreviations

53BP1: p53-binding protein 1
AAV: Adeno-associated virus
ATM: Ataxia-telangiectasia mutated proteins
BrdU: 5-Bromo-2′-deoxyuridine
C-Cas3: Cleaved-Caspase3
DAMPs: Damaged-associated molecular patterns
DSBs: Double-strand breaks
ELISA: Enzyme-linked immunosorbent assay
IF: Immunofluorescence
LBD: Lewy body dementia
NOR: Novel object recognition
PBS: Phosphate buffer saline
PD: Parkinson’s disease
PPARγ: Peroxisome proliferator activated receptor γ
SCT: Sucrose consumption test
Ser129: Serine-129
WB: Western blot
α-syn: Alpha-synuclein
